# Heterozygosity in an Isolated Population of a Large Mammal Founded by Four Individuals Is Predicted by an Individual-Based Genetic Model

**DOI:** 10.1371/journal.pone.0043482

**Published:** 2012-09-20

**Authors:** Jaana Kekkonen, Mikael Wikström, Jon E. Brommer

**Affiliations:** 1 Department of Biosciences, University of Helsinki, Helsinki, Finland; 2 Dotterböle, Tenhola, Finland; 3 ARONIA Coastal Zone Research Team, Novia University of Applied Sciences & Åbo Akademi University, Ekenäs, Finland; University of York, United Kingdom

## Abstract

**Background:**

Within-population genetic diversity is expected to be dramatically reduced if a population is founded by a low number of individuals. Three females and one male white-tailed deer *Odocoileus virginianus*, a North American species, were successfully introduced in Finland in 1934 and the population has since been growing rapidly, but remained in complete isolation from other populations.

**Methodology/Principal Findings:**

Based on 14 microsatellite loci, the expected heterozygosity H was 0.692 with a mean allelic richness (AR) of 5.36, which was significantly lower than what was found in Oklahoma, U.S.A. (H = 0.742; AR = 9.07), demonstrating that a bottleneck occurred. Observed H was in line with predictions from an individual-based model where the genealogy of the males and females in the population were tracked and the population's demography was included.

**Conclusion:**

Our findings provide a rare within-population empirical test of the founder effect and suggest that founding a population by a small number of individuals need not have a dramatic impact on heterozygosity in an iteroparous species.

## Introduction

A reduction in population size depletes genetic variation [1], [2], [3], [4], leading to an increased risk of local population extinction [5]. General population genetic theory predicts that a sudden reduction in effective population size leads to an exponential decay in heterozygosity with a loss rate determined by the effective population size [6], [7], [8]. When ignoring mutations,
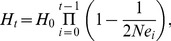
(1)where the heterozygosity at time *t* (*H_t_*) is given by the initial heterozygosity *H*_0_ of the founders declining with a rate inversely related to the time-specific effective population size *Ne_i_* [7],[8]. It has been recognised that this theorem can be oversimplistic when considering natural populations. For example, many populations have retained more of their diversity after a bottleneck than was predicted [9]. In particular, the predictions of classic theory (eq. 1) do not hold in organisms with overlapping generations (iteroparous organisms), because the loss of alleles is then much reduced [10]. In contrast to the classic theory, an individual-based population genetic model predicts that final heterozygosity is largely independent of the initial heterozygosity H_0_ in the founding population; heterozygosity may well increase after founding [11]. This is because when there are only few individuals, offspring heterozygosity critically depends on the details of the genetic composition of parents, since two homozygotes can produce heterozygous offspring if they are homozygous for different alleles. Despite its shortcomings, classic theory (eq. 1) is often applied also to organisms with overlapping generations by assuming a certain generation time (e.g. [12]). Unexpectedly high genetic diversities have, apart from overlapping generation, been attributed to migration [13] or to selection favoring heterozygosity itself (through overdominance or genetic incompatibility; [14], [15]). Invoking the latter explanation in the absence of detailed individual-based information clearly requires a solid basis of predicting the level of heterozygosity one would expect to find in a population.

The empirical evidence for a decline in heterozygosity with decreasing population size mostly concern cross-population comparisons. For example, there is a positive relationship between the size of a population and its genetic diversity across populations [3]. In contrast, the theory, as well as its implication, is typically set within a longitudinal framework of loss of heterozygosity over time in a specific population. It is therefore interesting to study the genetic consequences of a sudden reduction in population size in a population with known history. While the genetic consequences for bottlenecked populations are well studied [16],[17], tracking the consequences of founding a new population with few individuals is scarce. This is because it requires the founded population to have no secondary contact to its source or other population (i.e. no gene flow with other populations). Apart from increasing our fundamental understanding of the interaction between small population size and genetic diversity, such within-population studies may increase our knowledge of consequences of the introduction of organisms in the wild. Introducing an organism in nature in a locality where it currently does not occur and which migrants are unlikely to reach is anticipated to be an increasingly common feature in conservation biology [18]. Many species which are or which are expected to go extinct may be re-introduced in the future (e.g. from zoos), provided the cause of their extinction has since been amended. Introduction may also be carried within the context of “assisted migration”, where a species is introduced into new, suitable habitat which lies beyond an unsurpassable barrier [19]. From this perspective, studying the long-term population genetic consequences of a deliberate and documented past introduction is likely to provide useful insights for putative scenarios of species introductions in the future.

A population of the white-tailed deer *Odocoileus virginianus*, a North-American species, was founded in Finland in 1934 by three females and one male. A second introduction was attempted in 1948 ([20], Table 1, Table S1). There is no other population of white-tailed deer in the vicinity and the Finnish population has thus been living in island-like isolation from its source population in North America since its introduction. After being founded by a small number of individuals, the species has increased in numbers rapidly and expanded its range throughout most of the southern and central parts of the country. At present, Finland has a strong population of this species consisting of about 40 000–50 000 white-tailed deer. Thus, the white-tailed deer in Finland presents a potentially tractable case study on the extent of founder effects observable in a highly successful introduction. The objectives of this paper are to: (1), Document the details of the founding of this population based on the Finnish popular science literature of that time and other sources. (2), Quantify the genetic diversity, based on 14 microsatellite markers, of the current Finnish white-tailed deer population and compare this to published information on a North American population to evaluate the extent of the bottleneck. (3), Provide an exploration of theoretical predictions of heterozygosity and allelic richness in order to evaluate whether one can understand the observed level of genetic diversity in the current population on the basis of historical information on the founding of this population. To this end, we construct an individual-based population genetic model which incorporates what is known about the founding of the Finnish white-tailed deer and explore various scenarios of assumed genetic diversity of the founders.

**Table 1:** Short summary of available information on the development of the population of white-tailed deer in Finland in the establishment phase.

Year	N	Comment
0) 1934	5	4 f calves and 1 m calf arrive from Minnesota (U.S.A.) and are put inside an enclosure in Laukko estate.
3) 1937	6	Animals reproduce for the first time, inside the enclosure. Two females get 1 calf each (sex known). One of the four original females dies in autumn without having calved. This female is therefore known to not have left any descendants and is here not considered a founder of the population.
4) 1938	8	The same two females that reproduced in 1937 again produce 1 calf each (sex unknown). Animals are released from enclosure.
5) 1939	12	
7) 1941	15–20	
11) 1945	30–40	
14) 1948	90–100	3 f calves and 3 m calves arrive to Finland from U.S.A. and kept in enclosure
15) 1949		3 f 1-yr and 1 m 1-yr are released (2 male calves had died). The fate of the released animals is uncertain.
23) 1956	ca. 200	
27) 1961	ca. 1000	Hunting starts.

Population size (N) is an estimate of all individuals (calves, 1-year olds and adults) of both sexes. Information on population composition distinguishes female (f) and male (m) age groups. Detailed information on the establishment and available census statistics for the period 1934–1984 are provided in Table S1.

## Materials and Methods

### Founding of the Finnish white-tailed deer population

General information on the introduction and fate of the introduction of white-tailed deer in Finland and other places is described in [21]. We searched for specific information concerning the first years of the introduction in Finland and for estimates of population size by reading all the Finnish hunting magazines from the year of the introduction onwards. Until the outbreak of the Second World War, there was a general interest in the fate of the white-tailed deer population in Finland. Especially reports of gamekeepers involved with the establishment of the white-tailed deer were considered reliable.

### Collection of samples

Sampling of the white-tailed deer was done in south western Finland during two hunting seasons in winters 2009–2010 and 2010–2011. Samples were obtained by local hunters who shot the animals and collection involved several hunting groups operating over an area of approximately 400 km^2^ around Tenhola (60°3.5′N, 23°17.8′E). This area belongs to the core distributional area of the white-tailed deer in Finland. Hunters cut a small piece of meat and place it into a small plastic bag along with information sheet about the animal and time and location. No hunters were encouraged or financially rewarded to collect samples to be used in this study. White-tailed deer hunting in Finland is state regulated (Hunting Act of 1 August 1993 (615/1993) with subsequent amendments; unofficial translation to English, URL http://www.finlex.fi/en/laki/kaannokset/1993/en19930615.pdf). Hunting groups are allowed to cull a particular number of adult male, female and fawn white-tailed deer, the number of which is decreed by the Ministry of Agriculture and Forestry. All animals culled fell under the license provided to the hunting groups. Animals were culled on private land, under permission of the land owner. In the first winter 79 samples were collected and in the second winter 100.

### Microsatellite genotyping

DNA was extracted from a small piece of meat following the method described in [22], except that 70 µl of dH_2_O was used to elute DNA in the last step. The samples were amplified in Polymerase Chain Reaction (PCR) for twenty one microsatellite loci in four parallel panels. PCR was conducted with Phusion Flash master mix (Finnzymes), where one PCR reaction contained 5 µl of master mix-solution, 2 µl of extracted DNA, 1 µl of dH_2_O and 2 µl of primer mix. Panel 1 included primers INRA011 [23], Cervid1 [24], ILSTS011 (0.5 µM) [25], OCAM [26] and BovPRL (1 µM) [27]. Panel 2 included N, Q [28], ETH152 [29] and BM203 (0.5 µM) [30]. Panel 3 included K [28], BL25, BM6438, BM848 [30] and O (0.5 µM) [28]. Panel 4 included BM415 (1.0 µM), BM6506, BM4208 (0.5 µM) [30], R (1.0 µM), P, D [28] and OarFCB193 (0.5 µM) [31]. PCR's for all panels were completed on a BioRad S1000 Thermal cycler, using the following protocol (annealing temperature 58°C for panels 1 and 2 and 54°C for panels 3 and 4): one denaturing step of 10 s at 98°C followed by 30 cycles of 1 s at 98°C, 5 s at 58°C or 54°C depending on the panel, and 15 s at 72°C. Finally there was an additional 1 min at 72°C and an indefinite hold at 4°C. Forward primers were fluorescently labeled with FAM, HEX or TAMRA labels and PCR products were separated and visualized with an ABI 3730 sequencer (Applied Biosystems). Genotypes were scored using the software package Genemapper vs 4.1 (Applied Biosystems).

### Statistical analysis

The loci that were successfully amplified were checked for Hardy-Weinberg equilibrium and for linkage disequilibrium with software Genepop 3.4 [32].

We aimed to compare our results to a study from Oklahoma USA, which was based on 72 fully genotyped individuals [33]. We therefore only consider samples that had all the successful 14 loci fully genotyped (N = 80). To have exactly the same number of individuals as in the other study (N = 72), samples were randomly selected from our data. Inclusion of information on the genotypes of additional individuals available (n = 8) did not change the results. We therefore present the results for the 72 individuals for all analysis. After random selection there were 24 samples from winter 2009–2010 and 48 samples from winter 2010–2011. We compared the genetic diversity in our Finnish samples to that found for the same 14 loci in the North American study.

Expected heterozygosity and allelic richness were estimated using software FSTAT 2.9.3 [34]. Software BOTTLENECK 1.2.02 [35] was used to test whether the population has gone through a decline in population size in recent history. This was done by assuming the two-phase mutation model (TPM) as recommended for microsatellite data. The variance of mutations was set to 30 and the proportion of mutations larger than one step to 30%. Significance of the mismatch between the observed and expected heterozygosities was tested by using the Wilcoxon test and the visual graphic test [35]. Furthermore, another type of test for bottlenecks (Garza-Williamson test) was conducted in Arlequin 3.11 [36].

A non-parametric Mann-Whitney U-test test was carried out using the program SPSS 17.0 (SPSS for Windows, Rel. 17.0.0.2008, Chicago: SPSS Inc.) to test whether the white-tailed deer in Finland and in Oklahoma USA differed in expected heterozygosity or allelic richness.

### Individual-based model

We constructed a model which takes into account the demography and genealogy of the introduction of the white-tailed deer population. The model was based on tracking individual-specific genotypes in order to estimate expected heterozygosity across a number of scenarios. Because the composition of the founder population is not known, the initial population consisted of 3 females and 1 male with each 14 loci with an allelic richness equal to that observed in the population (see below for a scenario where this assumption was relaxed). Because information on the heterozygosity of founders is lacking, we considered both a scenario of maximal heterozygosity (H = 1) and one where heterozygosity was minimal given the number of alleles per locus. Individual-specific information on reproduction and survival was available for 1934–1938 and was included in the model in detail. Starting 1939, all females mated with a randomly-chosen male and reproductive output was assumed to follow age-specific probability values for producing 0, 1 or 2 fawns [37]. A newborns's genotype was the combination of two randomly chosen alleles at each locus, one from the mother and one from the father. Individual heterozygosity was estimated as the proportion of all loci that were heterozygous. Population-wide heterozygosity was the average heterozygosity across individuals. Survival was based on values that constrain the demography of the simulated populations to reflect the data on census population sizes. We assumed separate values for over-winter survival of a newborn (survival of 0.75), survival of a yearling (0.80) and survival of older age classes (0.85). Given these high survival rates, maximum lifespan was assumed to be sixteen years (under natural condition, maximum lifespan is thought to be 13 years, [38]). Density dependence was not included, because under the above presented parameter values, the demography of the population resembled the available census estimates (see Results) suggesting initial population growth after introduction was not constrained. Because reproduction and survival were probabilistic, except for the initial five years, the trajectory of population size varies because of demographic stochasticity between model runs. One thousand replicate population introductions were simulated in order to calculate the expected (i.e. mean) population sizes at various time steps as well as its lower and upper 95% confidence interval values. The model predicted the mean and 95% confidence interval (based on the 1000 replicates) of heterozygosity 45 years after the introduction (equivalent to the period 1934–1975), by which time the simulated population size was large (>1000) and heterozygosity did not change anymore. In some replicates, the population went extinct (because of demographic reasons) and these replicates were excluded. The individual-based population genetic model was a purpose-specific model (Programme S1) coded in MATLAB (The Mathworks, Inc., Massachusetts, USA).

Apart from the above described scenario (scenario A) with maximal and minimal heterozygosity of founders, we further explored two alternative scenarios. A bottleneck reduces the allelic richness per locus, especially for loci with high allelic richness. Hence, the current allelic richness is a minimum of what the allelic richness in the founders would have been. In scenario B, we therefore assumed allelic richness in the initial population was maximal (i.e. 8 alleles per locus) which entails that the heterozygosity of the founders was 1. In scenario C, a putative second introduction was implemented in the model by adding 1 male and 3 females, each with a completely heterozygous, unique and novel genotype, at time step 15 ( = 1949). See Text S1 for further details and rationale on model parameters and scenarios.

## Results

### Introduction of white-tailed deer in Finland

One male and four female white-tailed deer calves were released in Finland in 1934 and were kept in an enclosure until 1938. One female died before reproducing in 1937 and the maximal number of individuals in the first introduction therefore was four (1 male and 3 females). The four population founders were tame and the population stayed in the vicinity of the release site during the initial years. Detailed information of individual reproduction and survival was available during the period the animals were in the enclosure (Text S1). Estimates of population sizes were also available after release from the enclosure (Table 1, Text S1, Table S1). The population size estimates can be considered reasonable accurate at least during the first 1–2 decades (Table 1). However, population growth rate was probably high also after the 1960s (Table S1).

### Genetic diversity

Not all loci amplified successfully. Loci ILSTS011, OCAM, BovPRL, BM415, BM4208, R and P had a success rate of less than 10% and were not used. The remaining 14 loci were found to be in Hardy-Weinberg equilibrium and unlinked. Locus-specific values are presented in Table 2 and more detailed locus-specific information including allele frequencies in Table S4. Mean expected heterozygosity over loci was 0.692 (from 0.484 to 0.805). Mean number of alleles was 5.36 (from 2 to 8). When the results were compared to the published information on white-tailed deer of Oklahoma USA [33], smaller values for both diversity indices were found from the Finnish compared to the Oklahoma population (Mann-Whitney U-test A_R_: Z = −2.565, one tailed p = 0.0045; H_E_: Z = −1.654, one tailed p = 0.052). Based on results from the program Bottleneck the founding event was seen in the Finnish populations (Wilcoxon tests: one tailed p = 0.00003; Sign test: expected number of loci with heterozygosity excess = 7.88, but observed loci with heterozygosity excess = 14 with p = 0.00031). However, the graphic test gave a normal L-shaped distribution. Garza-Williamson gave a test statistic of 0.70 which is not under the threshold value (0.68) reported in the literature for a bottlenecked population.

**Table 2:** Basic population-level statistics of genetic variability in the Finnish population and the population from Oklahoma (N for both 72).

Locus	Finland		Oklahoma	
	A_R_	H_E_	A_R_	H_E_
Cervid1	6	0.719	14	0.847
INRA011	4	0.646	5	0.667
N	7	0.805	13	0.876
Q	7	0.777	15	0.861
ETH152	6	0.796	8	0.800
BM203	8	0.799	12	0.742
K	2	0.497	3	0.452
BL25	4	0.484	4	0.593
BM6438	4	0.678	9	0.790
O	3	0.543	4	0.509
BM848	6	0.748	10	0.829
BM6506	5	0.677	9	0.787
D	7	0.720	9	0.824
OarFCB193	6	0.797	12	0.809
Over loci	5.36	0.692	9.07	0.742

Allelic richness (A_R_) and expected heterozygosity (H_E_) are presented per locus and over loci.

### Predictions of an individual-based simulation model

**Figure 1:**
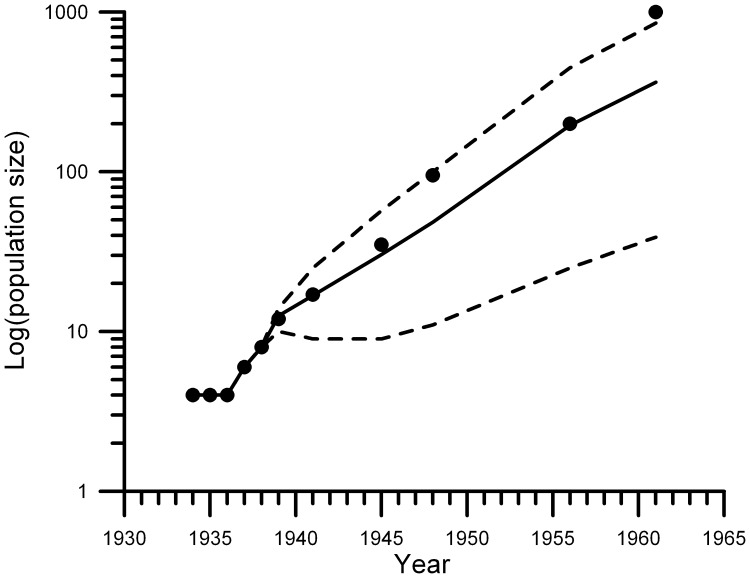
Temporal trend in population sizes (on 10-based logarithmic scale) of white-tailed deer adults and calves of both sexes after founding of the population. Plotted are both estimates found in the literature (dots) and the population sizes predicted by the individual-based population genetics model under the assumed vital and reproductive rates (Table S2). The solid line shows the average population sizes and the dotted lines the lower (2.5 percentile) and upper (97.5 percentile) of 1000 model replicates. The predicted population dynamics underlying all three founding scenarios (Table 3) are similar.

Under the chosen parameter values, the dynamics of the simulated population resembled the available information on the abundance of white-tailed deer after its introduction in Finland (Fig. 1). The white-tailed deer population clearly increased rapidly in size, essentially showing unhampered exponential growth during its establishment (solid line in Fig. 1). Because of this rapid growth, classic population genetic theory would predict that about 75% of initial heterozygosity can be maintained (Table S3; cf. [39]). Thus, if the founder population had the same initial heterozygosity as the Oklahoma population, expected H would be 0.55 on the basis of classic theory (Table S3), which is well below the observed H of 0.692.

In general, the population sizes predicted by the model under the assumed reproductive and vital rates closely resembled the estimates of white-tailed deer population available in the literature (solid dots in Fig. 1). However, the 1961 model-based numbers for population size were below the literature-based estimate of a population size of 1000 individuals (Fig. 1). Nevertheless, we consider the 1961 estimate of population size less reliable than estimates made in earlier years, because the white-tailed deer population was during these earlier years more restricted to the original site of release, and the population could therefore be censused more effectively. Only by assuming an unrealistic high survival or reproductive rate can the model-based population sizes reach 1000 individuals in 1961. Such large values would form a non-conservative assumption with respect to the loss of genetic diversity. We believe that the population dynamics predicted by our model captured the observed dynamics reasonably well.

The level of heterozygosity predicted by our scenario A closely resembled the observed value, irrespective of the initial heterozygosity (sub-scenarios A1 and A2; Table 3). The observation that predicted heterozygosity is independent from the heterozygosity of the founder population is the main difference between an individual-based model and the classic theory [eq. (1)]. The latter would predict that H = 0.74 if the founders had maximal heterozygosity, but predicted H = 0.29 when one assumes the founders had minimal heterozygosity (Table S3).

**Table 3:** Summary of the observed heterozygosity (H) and allelic richness (AR) of the white-tailed deer population and in individual-based population genetic simulations of its introduction.

Data	Sc.	H_0_	AR_0_	2^nd^ intr.	H	AR
Observed					0.679	5.36
Simulated	A1	1	5.36	No	0.631 (0.496–0.707)	4.40 (3.00–5.21)
	A2	0.39	5.36	No	0.630 (0.454–0.716)	4.38 (2.71–5.21)
	B	1	8	No	0.725 (0.555–0.802)	6.01 (3.57–7.57)
	C	1	5.36	Yes	0.636 (0.510–0.707)	4.50 (3.29–5.36)

Different scenarios (Sc.) were simulated. Scenario A simulated the introduction in 1934 of three females and one male of either maximal (A1) or minimal initial heterozygosity (A2) in 14 loci, assuming their allelic richness per locus is equal to that observed in the current population. Scenario B explores the consequences of maximal allelic richness ( = 8) per locus in the founding population (H_0_ must be 1). Scenario C explores the consequences of a successful second introduction (2^nd^ intr.) in 1949 of one male and three females, assumed to be all heterozygous and carrying novel alleles. All values are means and (in brackets), the 2.5- and 97.5-percentile of 1000 population replicates. Reported are the heterozygosity and allelic richness predicted by the model. Observed H was measured in 2009/2010 (Table 1). Vital rates were set to values which allowed the modelled population sizes to mimic the observed ones (Fig. 1).

Although scenario A predicted the heterozygosity of the population very well, it also predicted that the population would have a lower allelic richness than observed (Table 3). Nevertheless, under scenario A we assumed that the initial value for allelic richness was the allelic richness observed in the current population, which hence is the theoretical minimal value the founders could have had. Allowing the four founders to have the maximal allelic richness (i.e. 8 alleles per locus, which is our scenario B in Table 3) showed that both the observed heterozygosity and the observed allelic richness are consistent with the putative outcome of a single founding event. A similar result was obtained when relaxing scenario B's strong assumption of maximal allelic richness. Assuming that the initial allelic richness was only modestly higher than in the present population also produced a good fit to the observed heterozygosity and allelic richness (results not shown). Lastly, we explored the consequences of a successful second introduction for predicted heterozygosity and allelic richness (scenario C). We assumed that the second introduction consisted of fully heterozygous individuals with alleles that are all novel to the founded population. The effect on predicted heterozygosity of a successful second introduction event was minimal. Scenario C predicted that allelic richness was somewhat increased in comparison to scenario A and the upper confidence interval of the model-predicted allelic richness now included the observed allelic richness (Table 3).

## Discussion

We find that the current population of white-tailed deer in Finland has fairly high genetic diversity, despite the fact that this population was presumably founded by three females and one male and has remained in absolute isolation from other conspecific populations. Based on 14 microsatellite loci, we do find a clear indication that the Finnish population has lost alleles and evidence of a reduction in heterozygosity compared to published information on a concurrent population in Oklahoma U.S.A. (which we here assume to represent the source population). However, the difference in heterozygosity was minimal (0.742 in Oklahoma and 0.692 in Finland; implying 93% was retained), although this reduction was marginally significant. In terms of classic population genetic theory (eq. 1), this high level of retention of heterozygosity in a population presumably founded by four individuals is unexpected. Nevertheless, our individual-based simulation of population establishment where the genealogy of the males and females in the population are tracked allowed calculation of a predicted heterozygosity which closely matches the observed heterozygosity in the current Finnish white-tailed deer population. Because the introduced white-tailed deer population enjoyed rapid initial population growth, it spent a relatively short time period at a small size, which has allowed the retention of relatively much genetic diversity.

The main strength of our study is that it concerns a large mammal introduced from across the Atlantic. We can therefore be confident that the population has experienced no gene flow from another population and can exclude unmonitored re-stocking event of the introduced population. The only re-stocking on record consisted of four 1-year olds released in 1949. These individuals are thought to not have survived for a long period after their release [20]. Even if successful and conservatively assumed to consist of four individuals that were completely heterozygous with all novel alleles, this putative introduction still has minimal impact on predicted heterozygosity. This is because the population was rapidly growing in numbers and was, at the time of the second introduction, already estimated at 100 individuals. The alleles introduced by the second introduction therefore play a minor role and are much affected by drift since they are swamped by the alleles remaining after the main bottlenecking event of the initial years when population size was small. Nevertheless, this putative second introduction could have had a favourable impact on the population's allelic richness, especially if the animals in the second introduction carried (as we conservatively assume in our model) all new alleles. Our modelling scenarios are not exhaustive and certainly do not allow us to rule out that some or even all of the animals of the second introduction made a genetic contribution. Our simulations do show, however, that the observed values are consistent with a single founding event of four individuals, which thereby forms the most parsimonious explanation of the current genetic diversity in the Finnish white-tailed deer population. More conclusive evidence on the success of the re-stocking event could, for example, be obtained through the detection in the current population of loci with an allelic richness exceeding 8 (the theoretical maximal allelic richness of the four founding individuals). There is a clear scope for detecting loci with more than eight alleles, because several of the microsatellite loci we here consider have more than 8 alleles in the North American population. In Oklahoma 3 to 15 alleles were found per locus when studying the same 14 microsatellites in 72 individuals [33], but in consecutive studies with larger sample sizes more alleles were found: 4–19 alleles in 176–228 individuals [40] and 6–22 alleles in 368 individuals [41]. Even though we have a reasonable number of samples for this type of study, our sample size may not, however, be high enough to allow reliable detection of alleles occurring in very low frequency. For example, investigation of the allelic richness observed in the maximal sample size per locus available to us (typically >150 individuals; Table S4) shows evidence of one additional rare (frequency <1%) allele in two of the 14 loci, when compared to the allelic richness of the 72 individuals we here consider (although it should be noted that also in the larger dataset no locus has >8 alleles). A proper investigation of details involving rare allele frequencies would require the consideration of mutation in our model. Because the Finnish white-tailed deer population probably exceeded 10 000 individuals since the beginning of the 1970s (Table S1), it is possible that allelic richness increased due to mutation. Mutations may be particularly likely for a number of the more variable microsatellites we here consider. However, to the extent that our present sample adequately reflects genetic diversity in the population, model predictions are consistent with observed diversity also without invoking mutations.

A second caveat concerns our comparison of genetic diversity between Finland and Oklahoma U.S.A. The white-tailed deer introduced in Finland were from Minnesota. Since there was no published information on genetic diversity of white-tailed deer in Minnesota, we considered Oklahoma a good substitute. In Oklahoma (and in many other states of the U.S.A., [42]), the white-tailed deer was overharvested and consisted at its lowest point in 1916 of around 500 individuals but nowadays the Oklahoma statewide population is over 300 000 individuals [43]. In contrast, the white-tailed deer in Minnesota have not been dramatically reduced in abundance. Our comparison of genetic diversity in Finland and Oklahoma thus presents a conservative estimate of what the reduction in heterozygosity after founding could have been, because the genetic diversity of the non-bottlenecked population of Minnesota in 1934, when the founders were captured there, is likely to have been at least as high as in the current population in Oklahoma. Furthermore, we have sampled Finnish white-tailed deer ca. 200 km south of the original site of release (within the high-density part of the species' national range). White-tailed deer have limited dispersal [44] and it remains possible that genetic diversity is higher close to the original site of release. Lastly, we assume that the genetic diversity of hunted individuals captures the genetic diversity present in the population. The white-tailed deer hunt is regulated in Finland with emphasis on culling fawns and restrictions on the number of males culled, which is a hunting scheme that is unlikely to present strong selection on heterozygosity [45].

One technical aspect of our results concerns the inconsistency between the two statistical tests for the detection of bottlenecks which we here used. Given the founding history of the Finnish white-tailed deer, we expected to find clear statistical evidence of bottlenecks. However, only the program “Bottleneck” showed a clear signal of a population reduction in the past, whereas the Garza-Williamson index gave a value just above the threshold for a bottlenecked population. Nevertheless, when examining locus-specific indices, it is noteworthy that in seven out of fourteen loci the Garza-Williamson is clearly below this threshold. In addition, if a population starts to recover immediately after the bottleneck the index also recovers [46].

Classic population genetics predict an inevitable decrease in initial genetic diversity after founding. The largest difference between this classic approach and individual-based population genetic models, which can deal with overlapping generations and can include details of the genealogy, is that genetic diversity in the established population may actually exceed that of the founding population [11]. Furthermore, in species with overlapping generations, extinction risk of alleles due to drift is lower than in species without overlapping generations [47]. From a practical perspective, it is most noteworthy that when the heterozygosity of the founder population is not known, classic theory predicts a wide range of heterozygosities. In our case, predictions of classic theory varied from 0.29 to 0.74, depending on whether initial heterozygosity was assumed to be minimal or maximal and despite the fact that the Finnish white-tailed deer enjoyed a high population growth. In cases as ours, where the genetic diversity of the founders cannot be established, it is reassuring that the predictions of an individual-based population genetics model are insensitive to the initial heterozygosity such that useful predictions of the genetic consequences of an introduction event can be made even in the absence of knowledge of the initial genetic diversity.

Despite these recognised differences between classic and individual-based population genetic models, relatively few studies have taken an individual based approach to simulate extensively how diversity in populations can change. Examples include a simulation study of genetic diversity of copper redhorse (*Moxostoma hubbsi*), which has been conducted to investigate at which level the population size needs to be in the future to retain certain proportion of genetic diversity of today [48]. Further, a simulation of vole populations was used to couple a genetically explicit model and a dynamic landscape model to show the need to incorporate natural environmental processes in genetic models [49]. Nevertheless, these studies did not have the benefit of a historically known bottleneck or founder event. The study most comparable to ours was done by [11], who used an individual-based model to show that an isolated population of moufflons *Ovis aries* founded by only 2 individuals harbours a higher heterozygosity than predicted. Their hypothesis was that selection may have increased heterozygosity in this population above the predicted level. This study, however, only considered the consequences of the founders having variable (minimal or maximal) heterozygosity under the assumption that the founders had minimal (i.e. currently observed) allelic richness and did not study, as we did here, the consequences of founding a population with maximal allelic richness. Because loss of alleles leads to a reduction in heterozygosity [7], evaluation of the robustness of model predictions should consider both heterozygosity and allelic richness. A biological difference between the species is that the growth of the moufflon population was limited, with population sizes staying well below 1000 individuals because of repeated population crashes. In contrast, the white-tailed deer in Finland enjoyed almost unhampered initial population growth rate at a rate consistent with high survival and excellent reproductive success. Such fast population growth minimizes loss of genetic diversity. Indeed, assuming lower survival rates lowers the predicted heterozygosity, but produces expected trends in population size which fall below the observed trend (results not shown).

Based on our findings, we would argue that a (re-)introduction scheme of a large mammal, such as the white-tailed deer, should prioritise investing its likely limited resources in maximising the initial population growth of the introduced individuals. Prioritizing population growth could mean that financial resources are invested in restoring habitat, in providing the introduced individuals protection from predators or in providing them supplementary food rather than financially investing in introducing more individuals to increase the genetic diversity of founders. The case study of the introduction of the white-tailed deer in Finland clearly shows that a population founded by very few individuals can be highly successful provided it rapidly increases in size.

In conclusion, founding a population on the basis of few iteroparous individuals may have little consequences for its genetic diversity, at least in terms of heterozygosity. This may seem surprising on the basis of general population genetic theory [8], but this theory is, however, not developed for predicting the heterozygosity of iteroparous species. We show that an individual-based model can accurately predict the consequences for genetic diversity in a flexible manner largely independent on assumed initial heterozygosity. Our findings offer insights that can be used in designing or evaluating (re-)introductions of organisms in the wild.

## Supporting Information

Table S1 **Extended tabulated summary of available information on the development of the population of white-tailed deer in Finland.**
(DOCX)

Table S2 **Reproduction and survival values, as implemented in the individual-based genetic model.**
(DOCX)

Table S3 **White-tailed population development and application of classic population genetic theory (** **eq. (1)** **in the main text).**
(DOCX)

Table S4 **Locus-specific allele frequencies of the Finnish population, presented for both the 72 individuals of this study and for the maximal number of individuals available per locus.**
(DOCX)

Text S1 **Introduction of the white-tailed deer in Finland. Details of its introduction and further information on rationale behind model parameters.**
(DOCX)

Programme S1 **Zipped archive with MATLAB M-files forming the individual-based population genetics model used in this study to simulate the introduction of the white-tailed deer in Finland.**
(ZIP)
