# Supplementary material for: Heterozygosity in an Isolated Population of a Large Mammal Founded by Four Individuals Is Predicted by an Individual-Based Genetic Model
Source: PLoS One. 2012 Sep 20;7(9):e43482. doi: 10.1371/journal.pone.0043482 (PMC3447869; doi:10.1371/journal.pone.0043482)
Supplement: Table S1 — Extended tabulated summary of available information on the development of the population of white-tailed deer in Finland. (DOCX) [file pone.0043482.s001.docx]

**Table S1**. Extended summary of available information on the development of the population of white-tailed deer in Finland. The combination of two years (e.g. 1960/1961) refers to a particular winter. Population size (N) refers to autumn census (includes calves). Composition distinguishes male (m) from female (f) and various age groups whenever this information is available. A shorter version of this table with details relevant to the individual-based model is reported in the main text (Table 1).

**–––––––––––––––––––––––––––––––––––––––––––––––––––––––––––––––––––––––––––**Year N Composition Comment

–––––––––––––––––––––––––––––––––––––––––––––––––––––––––––––––––––––––––––1934 5 4 f calves / 1 m calf Animals inside enclosure in Laukko estate

1935 5 4 f 1-yr / 1 m 1-yr Animals inside enclosure. No rut, argued to be because animals are still in poor condition after transport from the US.

1936 5 4 f 2-yr / 1 m 2-yr Animals inside enclosure. Rut observed in November.

1937 6 2 m calves Animals inside enclosure. Two females get 1 calf each. One female without calf dies in autumn (NB: this female is therefore known to not have left any descendants and is here not considered a founder of the population).

3 f 3-yr / 1 m 3-yr

1938 8 2 ? calves Two females get 1 calf each. The one female without calf also had no calf in the previous year. Animals are released from enclosure.

2 m 1-yr

3 f 4-yr / 1 m 4-yr

1939 12

1941 15 – 20

1945 30 – 40

1948 90 – 100 3 f calves / 3 m calves arrive to Finland from U.S.A. and kept in enclosure

1949 Released: 3 f 1-yr / 1 m 1-yr (2 male calves had died). The fate of the released animals is uncertain.

1950/51 ≥100 Severe winter and 15-20% of animals died

1956 ca. 200

1960/61 After trial in 1958 (1 female culled), hunting started and 9 males were culled.

1961 ca 1000

1961/62 25 animals culled

1962/63 71 animals culled, mostly males

1963/64 123 animals culled, all adults

1964/65 311 animals culled

1965/66 ca. 2000 40% calves 491 animals culled

1966 2442 Inventory, includes calves. High density around point of release (100 animals / 1000 ha)

1966/1967 318 animals culled

1967/1968 536 animals culled

1968 ca. 4000 Census includes calves

1968/69 538 animals culled

1969/70 550 animals culled

1970/71 560 animals culled

1971/72 720 animals culled

1972/73 832 animals culled

1973 ≥ 10 000

1973/1974 1299 animals culled, but now also including several calves. 544 animals killed in traffic accidents.

1978 35 000 8640 animals culled

1980 42 000 15 000 animals culled

1981—83 Severe winter mortality. Low reproductive output in 1981.

1984 25 000 – 28 000

**–––––––––––––––––––––––––––––––––––––––––––––––––––––––––––––––––––––––––––**
