# Supplementary material for: Heterozygosity in an Isolated Population of a Large Mammal Founded by Four Individuals Is Predicted by an Individual-Based Genetic Model
Source: PLoS One. 2012 Sep 20;7(9):e43482. doi: 10.1371/journal.pone.0043482 (PMC3447869; doi:10.1371/journal.pone.0043482)
Supplement: Table S2 — Reproduction and survival values, as implemented in the individual-based genetic model. (DOCX) [file pone.0043482.s002.docx]

**Table S2**. Reproduction and survival as implemented in the individual-based genetic model. Reproduction was assumed to follow published information (Ryman et al. 1981), modelled as a set of Bernouille trials consisting of (1) the probability to produce at least one offspring (*p*_1_) followed by (2) if a female produces offspring, the probability she would produce not one but two offspring (*p*_2_). Values of age class greater than 8 are not presented by Ryman et al. (1981) and are inferred, assuming reproductive senescence. Survival (*s*) is presented for age *x* to *x*+1 with *l_x_* indicating the proportion of a cohort still alive at age *x*.

**–––––––––––––––––––––––––––––––––––––––––––––––––––––––––––––––––––––––––––**

Age Fecundity Survival

––––––––––––––– –––––––––––––

*p*_1,_*_x_* *p*_2,_*_x_* s_x_ l_x_

–––––––––––––––––––––––––––––––––––––––––––––––––––––––––––––––––––––––––––

0 0 0 0.75 1

1 0.05 0 0.8 0.75

2 0.75 0.733 0.85 0.64

3 0.80 0.75 0.85 0.57

4 0.90 0.667 0.85 0.49

5 0.90 0.667 0.85 0.41

6 0.80 0.625 0.85 0.35

7 0.70 0.429 0.85 0.30

8 0.50 0 0.85 0.25

9 0.50 0 0.85 0.22

10 0.50 0 0.85 0.18

11 0.40 0 0.85 0.16

12 0.40 0 0.85 0.13

13 0.30 0 0.85 0.11

14 0.30 0 0.85 0.10

15 0.20 0 0.85 0.08

16 0.10 0 0.85 0.07

**–––––––––––––––––––––––––––––––––––––––––––––––––––––––––––––––––––––––––––**
