# Supplementary material for: Heterozygosity in an Isolated Population of a Large Mammal Founded by Four Individuals Is Predicted by an Individual-Based Genetic Model
Source: PLoS One. 2012 Sep 20;7(9):e43482. doi: 10.1371/journal.pone.0043482 (PMC3447869; doi:10.1371/journal.pone.0043482)
Supplement: Table S3 — White-tailed population development and application of classic population genetic theory ( eq. (1) in the main text). (DOCX) [file pone.0043482.s003.docx]

**Table S3**. White-tailed population development and application of classic population genetic theory (eq. (1) in the main text). Classic theory works in steps of a generation time (T) and we assumed a generation time of 4 years. Census population size (N_c_) is based on data in Table S1. For the first generation (the founders), the effective population size (N_e_) is assumed to equal N_c_, but is after that assumed to be 70% of N_c_ (because calves and yearlings are part of the census). The heterozygosity (H) of each generation is calculated for three putative scenarios of H of the founder population. Initial H (H_0_) was either the same as in the USA (Oklahoma), maximal or minimal (see main text). Because of the high population growth rate, the predicted H quickly converges to its final value (Frankham et al. 2004).

**–––––––––––––––––––––––––––––––––––––––––––––––––––––––––––––––––––––––––––**

Year T N_c_ N_e_ Initial Heterozyosity (H_0_)

––––––––––––––––––––

USA maximal minimal

–––––––––––––––––––––––––––––––––––––––––––––––––––––––––––––––––––––––––––

1934 0 4 4 0.74 1 0.39

1938 1 8 5.6 0.65 0.88 0.34

1942 2 20 14 0.59 0.80 0.31

1946 3 40 28 0.57 0.77 0.30

1950 4 120 84 0.56 0.75 0.29

1954* 5 100 0.56 0.75 0.29

1958 6 200 140 0.55 0.75 0.29

1962 7 1000 700 0.55 0.74 0.29

**–––––––––––––––––––––––––––––––––––––––––––––––––––––––––––––––––––––––––––**

* Census not available, extrapolated value
