# Supplementary material for: Heterozygosity in an Isolated Population of a Large Mammal Founded by Four Individuals Is Predicted by an Individual-Based Genetic Model
Source: PLoS One. 2012 Sep 20;7(9):e43482. doi: 10.1371/journal.pone.0043482 (PMC3447869; doi:10.1371/journal.pone.0043482)
Supplement: Table S4 — Locus-specific allele frequencies of the Finnish population, presented for both the 72 individuals of this study and for the maximal number of individuals available per locus. (DOCX) [file pone.0043482.s004.docx]

**Table S4.** Locus-specific allele frequencies. For each locus (Anderson et al. 1992), the allelic frequency (A_Freq_) is presented both for the 72 samples that had all 14 loci successfully amplified and which were the focus of the comparison to USA (see main text) and for all individuals out of the full set of 172 samples, which were successfully genotyped for a particular locus. The number of individuals (N) for which each locus was successfully amplified is given for the latter dataset. For both sets of data locus-specific allelic richness (A_R_) and expected heterozygosity (H_E_) are reported. For two loci, there was an additional allele in the larger dataset, and the missing allele in the dataset with 72 individuals is then designated with frequency ‘N/A‘.

**–––––––––––––––––––––––––––––––––––––––––––––––––––––––––––––––––––––––––––**

72 individuals all individuals successful

––––––––––––––––––––––––– –––––––––––––––––––––––––

Locus Allele A_Freq_ A_R_ H_E_ A_Freq_ A_R_ H_E_ *N*

–––––––––––––––––––––––––––––––––––––––––––––––––––––––––––––––––––––––––––

Cervid1 6 0.719 6 0.713 *159*

175 0.375 0.355

177 0.340 0.365

179 0.056 0.028

181 0.049 0.063

185 0.028 0.035

189 0.153 0.154

INRA011 4 0.646 4 0.644 *158*

195 0.236 0.228

199 0.153 0.177

201 0.521 0.519

209 0.090 0.076

N 7 0.805 7 0.800 *156*

305 0.201 0.253

313 0.035 0.026

315 0.236 0.228

317 0.097 0.099

321 0.285 0.256

325 0.042 0.048

329 0.104 0.090

Q 7 0.777 7 0.750 *125*

241 0.076 0.068

245 0.104 0.088

247 0.007 0.004

257 0.243 0.276

273 0.104 0.100

275 0.361 0.384

283 0.104 0.080

ETH152 6 0.796 6 0.795 *166*

177 0.306 0.301

185 0.118 0.087

191 0.090 0.130

193 0.097 0.090

195 0.250 0.256

197 0.139 0.136

BM203 8 0.799 8 0.807 *167*

212 0.049 0.057

216 0.056 0.048

218 0.056 0.048

220 0.069 0.054

222 0.208 0.225

228 0.118 0.129

234 0.361 0.323

236 0.083 0.117

K 2 0.497 2 0.498 *173*

201 0.444 0.457

205 0.556 0.543

BL25 4 0.484 4 0.466 *174*

179 0.160 0.144

181 0.694 0.707

183 0.097 0.118

185 0.049 0.032

BM6438 4 0.678 4 0.665 *172*

263 0.035 0.017

271 0.417 0.419

273 0.250 0.244

275 0.299 0.320

O 3 0.543 3 0.556 *177*

188 0.243 0.232

196 0.618 0.605

204 0.139 0.164

BM848 6 0.748 6 0.759 *166*

372 0.403 0.389

376 0.174 0.160

378 0.118 0.108

380 0.208 0.202

382 0.090 0.120

386 0.007 0.021

BM6506 5 0.677 5 0.686 *167*

196 0.424 0.395

198 0.007 0.006

200 0.042 0.042

202 0.201 0.240

206 0.326 0.317

D 7 0.720 8 0.731 *169*

144 N/A 0.006

158 0.014 0.015

162 0.444 0.423

170 0.042 0.038

174 0.104 0.101

178 0.007 0.018

182 0.201 0.183

186 0.188 0.216

OarFCB193 6 0.797 7 0.778 *167*

100 N/A 0.003

102 0.097 0.057

118 0.097 0.105

120 0.188 0.141

122 0.139 0.147

124 0.340 0.371

126 0.139 0.177

**Over loci 5.36 0.692**

**–––––––––––––––––––––––––––––––––––––––––––––––––––––––––––––––––––––––––––**
