# Supplementary material for: Heterozygosity in an Isolated Population of a Large Mammal Founded by Four Individuals Is Predicted by an Individual-Based Genetic Model
Source: PLoS One. 2012 Sep 20;7(9):e43482. doi: 10.1371/journal.pone.0043482 (PMC3447869; doi:10.1371/journal.pone.0043482)
Supplement: Text S1 — Introduction of the white-tailed deer in Finland. Details of its introduction and further information on rationale behind model parameters. (DOCX) [file pone.0043482.s005.docx]

Text S1. Introduction of the white-tailed deer in Finland. Details of its introduction and further information on rationale behind model parameters.

Supplementary information belonging to the article “Heterozygosity in an isolated population of a large mammal founded by four individuals is predicted by an individual-based genetic model” by Jaana Kekkonen, Mikael Wikström, Jon E. Brommer.

*Introduction of the white-tailed deer in Finland*

The introduction of the white-tailed deer in Finland is well described in the Finnish literature. We here provide a brief summary. The essential parts of the development of the population is also summarised in Table S1.

In 1934, 13 white-tailed deer calves were caught around Virginia, Minnesota, U.S.A. and the strongest 7 (3 males and 4 females) were sent to Finland aboard the S/S Scanmail (Moilanen 1986). After a three week journey, one male and four female calves arrived in Helsinki 8th September 1934. Two male calves had perished during the journey and the one male calf was weakened so much it had to be carried of the ship (Moilanen 1986). The animals were immediately transported further to Laukko estate (Vesilahti), where they were placed in an enclosure of 3.5 ha (Sievänen 1939). The animals fared well in the enclosure and were tame (Sievänen 1939) and had a personal caretaker (Arvi Sievänen), who also recorded the initial fate of the animals. The male was called Uros. The females were called Jenny, Tilda and Fanni and the fourth female, which was blind, was called Soki (Sievänen 1935). Fanni was the largest and always followed Uros (Sievänen 1935). There was no rut in 1935 and thus no offspring produced in 1936 (Rikala & Ylänne 1937, Rikala 1938). Uros rubbed the velvet off his antlers for the first time in September 1936 (Rikala 1938) when he was already 2.5 years old, suggesting the stress of the relocation had delayed his development. A veterinary checked three of the four females in autumn 1936 and found they were capable of reproducing (Salmi 1949). The fourth female could not be caught (Salmi 1949). Probably this was the doe Fanni, because the individual not captured was reported to be staying close to the male Uros. In 1937, Jenny and Tilda each got a male calf, but Fanny and Soki did not (Sievänen 1937, Sievänen 1939). In autumn 1937, the blind female Soki was seriously injured (most likely by an eagle) and had to be put down (Sievänen 1939). Before calving in 1938, the population thereby consisted of 4-year old male Uros, four-year old females Jenny and Tilda, both with one male fawn, and the four-year old female Fanni without offspring. The 22^nd^ of March 1938 all white-tailed deer escaped from the enclosure (Sievänen 1939). All could be caught again, except the male Uros and it was therefore decided to set all free (Sievänen 1939). Jenny and Tilda each got a calf in summer 1938, but not Fanni. In autumn, the doe Fanni and the two 1.5 year old male fawns separated from the group (Salmi 1949). Thus, the fate of the doe Fanni, which is the female known to, until that time, not have reproduced, is uncertain. The animals started to move some distances from the site of release. For example, Uros was spotted 8 km away (Sievänen 1939), but they regularly returned to the site of release.

Summer 1939, Arvi Sievänen counted five new calves among those animals staying close to Laukko, i.e. the population consisted of at least 12 individuals of which 7 were adults (Sievänen 1939). In 1940, the total number of animals was estimated at close to 20 and now included also twin calves (Sievänen 1941). The population grew rapidly. Hunting started in 1960 and also the hunting statistics reveal a rapidly growing population, apparently not restricted in its growth rate (Table S1).

*Re-stocking event of 1948*

Three calves arrived in Finland in 1937 and records indicate the animals were housed in the Korkeasaari Zoo (Helsinki). These animals were never introduced in the wild and disappeared during the following years, i.e. during the Second World War (Nygren 1984). There is little detailed information on the animals that were involved in the second introduction of 1948 in Laukko. Three female and three male calves arrived in 1948 and were kept one year in the enclosure. Two male calves died shortly after arrival and the surviving male and the three females were released from the enclosure the next year. No detailed information is available, but gamekeepers reported high mortality of calves and juveniles due to predation by eagles in the consecutive years (Nygren pers. comm.).

*Details on the individual-based simulation model*

Because one female died before producing offspring, we considered one male and three female founders. We assumed 14 loci per individual with allelic richness as observed in the study population (Table 2). In the basic simulations (scenario A), the initial population therefore consisted of one male and three females, whose genotypes were constructed such that they together included the above described allelic richness. Heterozygosity was either maximal (=1, scenario A1) or minimal given the locus-specific allelic richness (=0.385, in scenario A2) and individual heterozygosity was randomly assigned across the four founding animals. Because allele frequencies were not known, each allele at a locus was assumed to be equally common. Starting at t = 0 (1934), reproduction and survival in the first four time steps followed exactly what is known about the population (Table S1). That is, at t = 3, (1937), two randomly chosen females produced one son each. At t = 4 (1938), the same two females as in the previous time step produced two offspring of randomly determined sex (assuming equal sex ratio). Offspring genotypes were a random combination of half the male and half the female’s genotype. All animals survived to t = 5 (1939). From t = 5 onwards, reproduction and survival of individuals was simulated. Each female was paired to a randomly chosen (with resampling) male. This assumption ignores the mating system of the white-tailed deer, where male mating success is likely to be skewed. Ignoring this skew increases the effective population size and is thus likely to increase the predicted heterozygosity.

Survival was modelled as age-specific survival until the next time step (year) and was assumed to be potentially different for calves, 1-year olds and 2+-year olds, where the younger age classes were assumed to have lower survival. Parameter values for survival were adjusted to produce a trend in population size which encompassed the observed trend in population size. Under the assumed survival values (Table S2), assumption of approximately 16 age classes was deemed reasonable (ca. 6% of the population reaches age 16, Table S2). Note, however, that assuming maximally ten age classes produces qualitatively the same results, but requires a stronger truncation of the assumed life history (Table S2). Clearly, different combinations of survival values can be constructed to produce a reasonable fit to the observed population size, but in general survival values need to be high (s_0_ > 0.7, other s > 0.8) to produce a decent fit and different combinations of survival values produced qualitatively the same result (results not shown).

Reproduction was assumed to follow published information (Ryman et al. 1981), modelled as a set of Bernouille trials consisting of (1) the probability to produce at least one offspring (*p*_1_) followed by (2) if a female produces offspring, the probability she would produce not one but two offspring (*p*_2_). Values were age-specific, assuming a senescent decline (Table S2)

Population census in the model was carried out after reproduction and thus included individuals of all age classes (including calves).

Scenario B: The founding event causes an immediate reduction in allelic richness since the maximal allelic richness per locus is twice the number of founders. However, additional alleles may get lost from the population over time. Loci with high allelic richness are especially likely to lose alleles. This means that assuming the founders have the same allelic richness as what is observed in the current population may be misleading. Some of these alleles may get lost and predicted heterozygosity (based on, on average, a lower allelic richness than observed) may thus be an underestimate. To explore this scenario, we assumed that the founders had maximal allelic richness (i.e. 8).

Scenario C: The second introduction is assumed to have been unsuccessful, but detailed information on the fate of these animals is not available. We explored the predicted consequences of a potentially successful second introduction by assuming that the one male and three female entered the population at t = 15 (= 1949). Given the well documented negative impact of the translocation on the original (1934) founders (see above), it is likely that also the animals included in the re-stocking event had a poor performance. We therefore assumed a one-year delay in development and modelled the re-stocking as the release of zero-year olds at t = 15, which after that followed the assumed reproduction and survival values (Table S2).

References included in the Supplement and not in the main text

Moilanen, P. 1986. Valkohäntäpeuran tuontihistoria. In *Valkohäntäpeura, elintavat metsästys riistanhoito* (ed Moilanen, P. & Vikberg, P.), pp 15-29. Otava, Helsinki.

Moilanen, P. 1986. Valkohäntäpeurojen kannan kehitys Suomessa. In *Valkohäntäpeura, elintavat metsästys riistanhoito* (ed Moilanen, P. & Vikberg, P.), pp. 53-65. Otava, Helsinki.

Niemelä, E. 1986. Valkohäntäpeurojen metsästäminen. In *Valkohäntäpeura, elintavat metsästys riistanhoito* (ed Moilanen, P. & Vikberg, P.), pp. 66-75. Otava, Helsinki.

Rikala, K. & Ylänne, Y. 1937. Lisätietoja maamme kuusi- ja punapeuroista. *Metsästys ja Kalastus*, 178-180.

Salmi, A-P. 1948. Valkohäntäpeurojen esiintyminen ja elinmahdollisuudet Suomessa. *Metsästys ja Kalastus*, **7-8**, 193-197.

Salmi, A-P. 1949. Valkohäntäpeura Suomessa. *Suomen Riista* **4**, 91-124.

Sievänen, A. 1935. Tietoja Laukon punapeuroista. *Metsästys ja Kalastus* **3-1935**, 104.

Sievänen, A. 1937. Laukon punapeurat. *Metsästys ja Kalastus*, 287-288.

Sievänen, A. 1939a. Laukon peurat vapaudessa. *Metsästys ja Kalastus,* **3-1939**, 75-77.

Sievänen, A. 1939b. Ajokoirametsästys ja hirvieläimet – Asia Laukon peurojen kannalta katsottuna. *Metsästys ja Kalastus* **11-1939**, 338.

Sievänen, A. 1941. Laukon peuraa vedettiin kelkalla. *Metsästys ja Kalastus* 2-1941, 63.

Ylänne, Y. 1943. Maahamme tuotetut peuralajit. *Metsästys ja Kalastus*, **3-1943**, 94-95.

Ylänne, Y. 1947. Virginia-peuroja. *Metsästys ja Kalastus*, **3-1947**, 96.
